# Towards the implementation of a DNA barcode library for the identification of Peruvian species of *Anastrepha* (Diptera: Tephritidae)

**DOI:** 10.1371/journal.pone.0228136

**Published:** 2020-01-31

**Authors:** Ida Bartolini, Julio Rivera, Norma Nolazco, Arturo Olórtegui

**Affiliations:** 1 Laboratorio de Biología Molecular, Servicio Nacional de Sanidad Agrícola, La Molina, Lima, Perú; 2 Unidad de Investigación en Entomología y Medio Ambiente, Universidad San Ignacio de Loyola, La Molina, Lima, Perú; 3 Laboratorio de Entomología del Centro de Diagnóstico de Sanidad Vegetal, Servicio Nacional de Sanidad Agrícola, La Molina, Lima, Perú; Nanjing Agricultural University, CHINA

## Abstract

The genus *Anastrepha* is a diverse lineage of fruit-damaging tephritid flies widespread across the Neotropical Region. Accurate taxonomic identification of these flies is therefore of paramount importance in agricultural contexts. DNA barcoding libraries are molecular-based tools based on a short sequence of the mitochondrial COI gene enabling rapid taxonomic identification of biological species. In this study, we evaluate the utility of this method for species identification of Peruvian species of *Anastrepha* and assemble a preliminary barcode profile for the group. We obtained 73 individual sequences representing the 15 most common species, 13 of which were either assigned to previously recognized or newly established BINs. Intraspecific genetic divergence between sampled species averaged 1.01% (range 0–3.3%), whereas maximum interspecific values averaged 8.67 (range 8.26–17.12%). DNA barcoding was found to be an effective method to discriminate between many Peruvian species of *Anastrepha* that were tested, except for most species of the *fraterculus* species group, which were all assigned to the same BIN as they shared similar and, in some cases, identical barcodes. We complemented this newly produced dataset with 86 published sequences to build a DNA barcoding library of 159 sequences representing 56 Peruvian species of *Anastrepha* (approx. 58% of species reported from that country). We conclude that DNA barcoding is an effective method to distinguish among Peruvian species of *Anastrepha* outside the *fraterculus* group, and that complementary methods (e.g., morphometrics, additional genetic markers) would be desirable to assist *sensu stricto* species identification for phytosanitary surveillance and management practices of this important group of pestiferous flies.

## Introduction

Insect pests decrease crop productivity and restrict the exportation of agricultural products in optimal marketable conditions [1]. Fruit flies of the family Tephritidae are among some of the most aggressive insect pests, as they compete against humans for various types of ripening fruits; outbreaks may result in economic losses, crop destruction and international phytosanitary barriers [2]. More than 4900 species of tephritid fruit flies are currently recognized worldwide (A. L. Norrbom pers. comm.), of which about 200 are notorious for their noxious effects on crops, and thus are considered quarantine pests of economic importance at a global scale [3].

Various species within the genera *Bactrocera*, *Zeugodacus*, *Ceratitis*, *Rhagoletis*, *Anastrepha*, and a number of other genera, are particularly damaging for agriculture. For instance, only in Brazil *Ceratitis capitata* (Wiedemann) is responsible for US$242 million/year in economic losses [4]. Because of their threat to agricultural development, the Servicio Nacional de Sanidad Agrícola (SENASA) of Peru launched a special fruit fly program targeting this plague two decades ago. However, the complex geography, ecological diversity and vertical stratification of Peru proved to be challenging for the effective control of these insects, making necessary the implementation of multiple strategies to deal with the various problems resulting from targeting such diverse and versatile group of insects.

DNA barcoding is a tool conceived to inform taxonomic decisions. The method consist of isolating and sequencing a small fragment of the mitochondrial gene Cytochrome Oxidase I (COI). The COI DNA barcode is well conserved at the intraspecific level, thus allowing the segregation of species, and populations within species, but variable enough to result in genetic divergence gaps, defining interspecific boundaries [5]. Because accurate species identification is instrumental for effective pest eradication programs [6], DNA barcoding is becoming a broadly used technique in phytosanitary contexts. Given the great diversity of tephritid fruit flies and the broad range of crops they attack, accurate taxonomic identification is therefore particularly relevant for the fruit flies of the genus *Anastrepha*. Currently 96 species of the genus have been reported to occur in Peru, but with new ones being routinely discovered [7, 8, 9, 10, 11, 12] and many more pending description, the number of Peruvian species of *Anastrepha* is expected to increase well over 100 spp. (A. L. Norrbom pers. comm.). Incorporating DNA barcoding as customary method could thus help advance ongoing taxonomic studies on *Anastrepha* in Peru. The fact that seven of the nine species of *Anastrepha* considered major pests, namely *A*. *striata* Schiner, *A*. *obliqua* (Macquart), *A*. *serpentina* (Wiedemann), *A*. *grandis* (Macquart), *A*. *curvicauda* (Gerstaecker) (formerly *Toxotrypana curvicauda* Gerstaecker: see Norrbom et al., 2018[13]), and the infamous *A*. *fraterculus* (Wiedemann) complex, occur in this country [14], the need for efficient, reliable and cost-effective methods for their rapid taxonomic identification is a high priority.

The objective of this study is to evaluate the utility of DNA barcoding for species identification of Peruvian species of *Anastrepha* by assembling a preliminary barcode profile for the group using both newly generated and publicly available sequences. The information herein produced will set foundations for the implementation of a comprehensive DNA barcode library for the identification of *Anastrepha* fruit flies in Peru. This study also aims to identify potential caveats and limitations of this tool to set the course of future studies oriented to expand our knowledge on the diversity of these flies in Peru.

## Material and methods

### Specimens and geographic coverage

Voucher specimens selected for DNA extraction are deposited in the insect reference collection of the Laboratory of Entomology, Servicio Nacional de Sanidad Agrícola (SENASA), located in Lima, Peru. All specimens used for tissue extraction were collected in different localities across the country using McPhail traps between the years 1995 and 2012. All collected samples were preserved in 95% EtOH and taxonomically identified at SENASA by NN. Best preserved specimens were selected for tissue extraction and photographic documentation. All relevant voucher data was registered in the Barcode of Life Data Systems (BOLD) from which a dataset was assembled for the present study (BOLD project name: DS-TEPHPER). A spreadsheet summarizing relevant data (specimens, collecting sites, dates, etc.) can be found in S1 File. Specimens came from 19 of the 24 departments within Peru—the classic political subdivisions of the country—and from various ecological regions, including the northern and central Pacific coast (6 departments), the central and southern Andean highlands (8 departments), and northeastern and southeastern Amazon (5 departments).

### DNA extraction, public data and analyses

A single leg from each selected specimen was removed to obtain fresh tissue for DNA extraction. Sampled tissues were placed in microplates with wells containing absolute EtOH. All subsequent steps, including tissue lysis, DNA extraction, PCR amplification of segment 1 of the Cytochrome Oxidase I gene (COI), and sequencing, were carried out at the Canadian Center for DNA Barcoding (CCDB) facilities in Guelph (Ontario) using standard DNA barcoding protocols [15]. PCR amplification of the target genetic marker used the primer cocktail C_LepFolF (LepF1/ LCO1490) and C_LepFolR (LepR1/HCO2198). All COI sequences and associated information is available in BOLD under project DS-TEPHER (dx.doi.org/10.5883/DS-TEPHPER) and released to GenBank (Accession numbers MN454412–MN454491). We implemented the various functions available in the online workbench platform of BOLD (http://www.boldsystems.org) to analyze this dataset. We favored a minimalistic approach for data analysis in order to establish a methodological baseline for further efforts to build a comprehensive DNA barcoding library for Peruvian *Anastrepha* spp. Standard analytical functions therefore included “Taxon ID Tree”, “Distance Summary”, “Barcode Gap Analysis” and “BIN discordance”. Parameters of interest included the Kimura-2 parameter nucleotide substitution and the Muscle algorithm for automatic sequence alignment. To complement our dataset we obtained 86 additional sequences representing 41 Peruvian species of *Anastrepha*. These sequences were published elsewhere [16, 17, 18, 19] and are available at BOLD. Downloaded sequences were latter integrated to our original DNA barcoding dataset to produce a more comprehensive DNA barcoding tree profile for all Peruvian species of *Anastrepha* available up to date (associated alignment is available as supplementary material).

## Results and discussion

We sampled 168 individual tephritid flies, 73 of which (43.4%) yielded useful sequences, with 95% of sequences containing more than 600 bps (typically, 658 bps). The following outgroup taxa were included to root the resulting species tree (no. of specimens in parenthesis): *Bactrocera dorsalis* (Hendel) (formerly *Bactrocera papayae* Drew & Hendel) (4), *Trupanea metoeca* (Hendel) (1) and *Ceratitis capitata* (3). Mean nucleotide frequency distribution showed a bias towards A+T content (67.16%) vs C+G content (32.84%), values close to average percentages estimated for the COI gene of insects (i.e. ~ 69% for A+T, and ~ 31% for C+G contents) [20]. No stop codons were detected.

Analyses of sequences using the workbench platform implemented in BOLD recognized 16 species of *Anastrepha* distributed in 14 BIN’s, one of which corresponded to samples of *A*. *ludens* (Loew) from Mexico included for comparative purposes. The remaining 15 species of *Anastrepha* analyzed represent approximately 24% of the about 62 species reported for Peru ([7, 8, 9, 10, 12].

Mean and maximum global values of intraspecific genetic divergence within sampled species of *Anastrepha* were 1.01 and 3.3% respectively. The mean value of genetic divergence within *Anastrepha* was 8.67%, the lowest genetic divergence between species pairs from different species groups ranged from 8.26% to 8.97% in the case of *A*. *striata* (*striata* group) vs *A*. *serpentina* (*serpentina* group), and the highest divergence was between *A*. *nolazcoae* Norrbom & Korytkowski (*mucronata* group) and *A*. *grandis* (*grandis* group), ranging from 16.25% to 17.12%.

**Fig 1:**
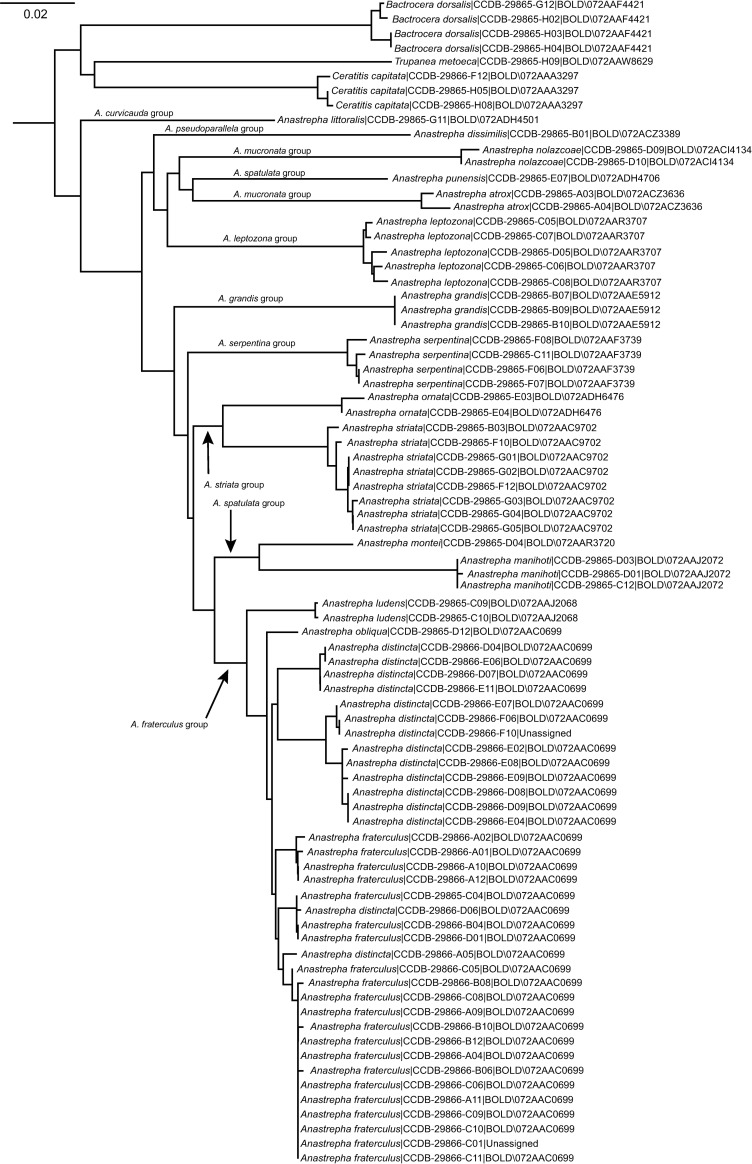
DNA barcoding tree of Peruvian *Anastrepha* spp. A Kimura 2-parameter Neighbor joining tree for 16 sampled species (and species groups) of *Anastrepha* showing their DNA barcoding profile. All species of *Anastrepha*, except for the two sequences of Mexican *A*. *ludens*, were derived from specimens collected in Peru (see S1 Table for specific localities). Labels on each species represent specimen´s identifier in BOLD (CCDB code), followed by their assigned BIN. Sampled Peruvian members of the *fraterculus* group shared the same BIN (AAC0699), making them difficult to segregate using the COI barcode molecular marker.

The species tree (Fig 1) resolved most of the ten sampled species groups independently, four of which had multiple species. Only the *mucronata* group did not form a single cluster; its two included species (*A*. *nolazcoae* and *A*. *atrox*) clustered with *A*. *punensis* (*dactiformis* group) with *A*. *atrox* (Aldrich) closer to *A*. *punensis* Tigrero & Salas. The other mixed cluster was the one formed by three closely related members of the *fraterculus* group, namely *A*. *fraterculus*, *A*. *distincta*, and *A*. *obliqua*. These three species were all assigned to the same BIN (AAC0699; see Barr *et al*. [19]), evidencing a close relationship. Members of this species group exhibited up to 25% of sequence overlap, and even identical or near identical barcodes in some cases (e.g., between *A*. *distincta* Greene and *A*. *fraterculus*). The max. values of intraspecific divergence within the group was estimated to be 3.3% for *A*. *distincta* sequences, the latter also found to be the nearest species to *A*. *fraterculus* with as little as 0.16% of divergence between some samples. Tested samples of *A*. *ludens* from Mexico, also belonging to the *fraterculus* group, clustered in their own BIN (AAJ2068) as expected, confirming them as genetically distinct from other members of the *fraterculus* group.

The maximum values of intraspecific genetic divergence for *A*. *nolazcoae* and *A*. *atrox* were, respectively, 0.49 and 1.12%, whereas *A*. *punensis* was a singleton. Within the *spatulata* group interspecific genetic divergence between *A*. *manihoti* Lima (max. intraspecific 0.15%; BIN: AAJ2072) and *A*. *montei* (singleton; BIN: AAR3720) ranged from 7.9 to 8.1%, whereas between *A*. *ornata* Aldrich (max. intraspecific 0.61%; BIN: ADH6476) and *A*. *striata* (max. intraspecific 1.23%; BIN: AAC9702), both in the *striata* group, interspecific values ranged from 6.28 to 7.59%. In *A*. *leptozona* Hendel we found a maximum intraspecific value of 0.92%. The remaining tested species had 0% of divergence, namely *A*. *grandis* (3 specimens sharing the same haplotype), *A*. *obliqua*, *A*. *dissimilis* Stone, and *A*. *littoralis* (Blanchard) (all singletons). Table 1 summarizes levels of genetic divergence (distance) and distance to Nearest Neighbor for this dataset.

**Table 1:** Levels of genetic divergence in *Anastrepha* spp.

Species	Mean % Intra-Sp	Max % Intra-Sp	Nearest Species	Nearest Neighbor (NN)	Distance to NN %
*A*. *atrox*	1.12	1.12	*A*. *distincta*	SENTO545-17	10.06
*A*. *dissimilis*	N/A	0	*A*. *distincta*	SENTO545-17	9.13
*A*. *distincta*	1.89	3.3	*A*. *fraterculus*	SENTO512-17	0.16
*A*. *fraterculus*	0.7	1.69	*A*. *distincta*	SENTO517-17	0.16
*A*. *grandis*	0	0	*A*. *distincta*	SENTO545-17	8.01
*littoralis*	N/A	0	*A*. *distincta*	SENTO545-17	9.07
*A*. *leptozona*	0.73	0.92	*A*. *distincta*	SENTO545-17	7.97
*A*. *ludens*	0.15	0.15	*A*. *fraterculus*	SENTO476-17	2.65
*A*. *manihoti*	0.1	0.15	*A*. *montei*	SENTO420-17	7.93
*A*. *montei*	N/A	0	*A*. *fraterculus*	SENTO500-17	4.76
*A*. *nolazcoae*	0.49	0.49	*A*. *distincta*	SENTO545-17	12.11
*A*. *obliqua*	N/A	0	*A*. *fraterculus*	SENTO504-17	1.54
*A*. *ornata*	0.61	0.61	*A*. *fraterculus*	SENTO500-17	6.25
*A*. *punensis*	N/A	0	*A*. *distincta*	SENTO545-17	9.9
*A*. *serpentina*	0.54	1.07	*A*. *fraterculus*	SENTO500-17	6.63
*A*. *striata*	0.47	1.23	*A*. *fraterculus*	SENTO500-17	5.19

Results of DNA barcode analysis showing min and max values of intraspecific divergence between species corresponding to dataset in Fig 1 and their nearest species, represented by the Nearest Neighbor (NN) sequence. Singletons are labeled as N/A and thus could not be evaluated.

**Fig 2:**
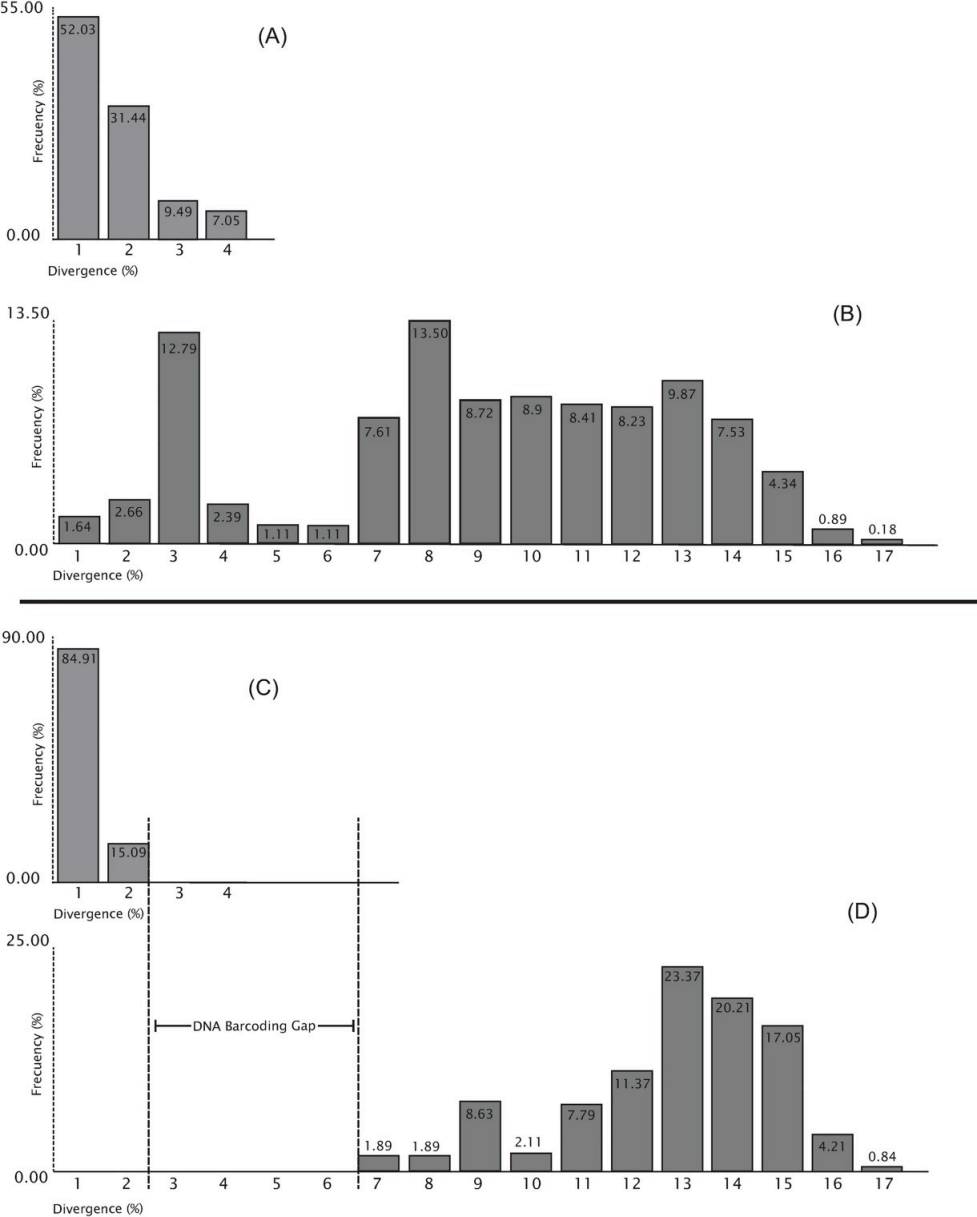
Genetic divergence Peruvian *Anastrepha* spp. Histograms showing levels of genetic divergence and their frequency (both in %): A and B represent intra- and interspecific distances respectively, as estimated on the dataset produced for this study (Fig 1), whereas C and D represent the same but excluding the *A*. *fraterculus* species group. The removal of the latter increases identification accuracy in about 16.5%. This demonstrates the existence of a DNA barcoding gap outside *fraterculus* group, and suggests that taxonomic identification through DNA barcodes may be feasible for the remaining Peruvian *Anastrepha* spp.

If we set aside *fraterculus* group data, we found that mean and max. intraspecific distance in the remaining *Anastrepha* species were 0.49% and 1.23% respectively, whereas at the interspecific level, min., mean and max. levels of genetic divergence were, respectively, 6.29, 12.34 and 17.12%. The removal of the *fraterculus* group data increased the accuracy of taxonomic identification (Fig 2). The min. value indicated above (i.e., 6.29%) also represents the distance to Nearest Neighbor (NN), corresponding to the divergence between *A*. *ornata* and *A*. *striata* (NN barcode SENTO395-17). This shows that a barcode gap, well above the 2% cut off proposed, exists among sampled species when members of the *fraterculus* group are excluded from the analysis (Fig 2), thus enabling molecular identification outside the *fraterculus* group. Our analysis also resulted in the identification of three new BINs: ADH6476 (*A*. *ornata*), ACZ3389 (*A*. *dissimilis*), and ADH4501 (*A*. *littoralis*). All remaining tested species are consistent with published BINs [16, 17, 18, 19] (see S1 Table).

Overall, DNA barcoding proved to be an effective method to discriminate between Peruvian species of *Anastrepha* (Fig 1), with limited effectiveness in members of the *fraterculus* group. Smit *et al*. [21] provides a useful baseline of DNA barcodes across Tephritidae, and thus we use their data as an initial approach for global comparison of our results. Smit *et al*. [21] sampled 135 species in 42 genera of Tephritidae across Europe and found that intraspecific genetic distance ranged from 0 to 2.8% (mean 0.24%). These figures are comparable to the maximum value of divergence we found in our dataset (excluding the *fraterculus* group): 1.23% (mean 0.49%). Smit *et al*. [21] estimated 0.15 to 25.27% (mean 13.2%) of divergence between species pairs, although a small percentage of these pairwise comparisons (about 2.7%) ranged between 0.15–2.8%, thus evidencing the lack of a barcoding gap in this subgroup (prominently represented by *Urophora* spp.). Nevertheless, at the intrageneric level Smit *et al*. [21] found divergences of up to 8.78% (mean 1.49%), whereas in our single-genus dataset these values were comparatively higher, up to 17.12% (mean 11.88%), evidencing a rich genetic diversity in the barcoding gene within *Anastrepha* alone. Overall, the values of genetic divergence in the DNA barcoding gene of Peruvian *Anastrepha* fall within known ranges reported for the family.

Within the genus *Anastrepha*, levels of intraspecific genetic divergence of all major pest species herein tested with no-zero values (namely *A*. *striata*, *A*. *serpentina* and the *A*. *fraterculus* group), were comparable to those obtained in Barr *et al*. [19], the most comprehensive source of DNA barcoding data for the genus to date. The intraspecific genetic divergence of 1.23% in *A*. *striata* contrasts with that of Barr *et al*. [17/19], who reported a maximum value of intraspecific genetic divergence of 0.9% for a sample of 18 sequences/specimens of *A*. *striata* from localities encompassing a much wider geographic distribution (from northeastern Mexico to the Amazon). Similarly, Gallo-Franco *et a*l. [22] found a maximum divergence value of 0.4% in *A*. *striata* across localities in Colombia. A similar pattern was observed in *A*. *serpentina*, which clustered under its reported BIN (AAF3739) with maximum intraspecific value of 1.07%, somewhat lower than 1.5% as reported in Barr *et al*. [19] for a sample of 30 sequences/specimens from localities ranging from northeastern Mexico to southeastern Brazil (one from Cusco, Peru), a value likely representative of the entire gene pool of this species. In contrast, our sampling of *A*. *serpentina* included only 5 specimens from geographic and ecologically scattered localities across Peru (Amazonas, Tumbes Junín, Lima and Cusco), and yet important genetic diversity was found within this country alone.

Our results pertaining to the *A*. *fraterculus* group are consistent with Barr *et al*. [19] in that all species tested (excluding of *A*. *ludens*), namely *A*. *fraterculus*, *A*. *distincta* and *A*. *obliqua*, were not resolved (Fig 1). The *A*. *fraterculus* group comprises 34 closely related species, 15 of which are indistinguishable through the DNA barcoding marker, as they all share the same barcode (BIN: AAC0699) (Barr *et al*., [19]). We found similar results in our sample of the *fraterculus* group, and thus our analysis confirmed the results of previous studies revealing the complex genetic background of members of the *A*. *fraterculus* group [23, 24]. Although the reasons for this are not thoroughly understood, Scally *et al*. [17] found evidence of mitochondrial introgression as a likely mechanism responsible for the existence of shared haplotypes among the members of this economically important group of tephritid flies, many of which are sympatric and widely distributed. The ability to adapt to a wide variety of ecological conditions and host crops seemingly offers plenty of ecological opportunities for hybridization to occur naturally in these flies [25].

Overcoming these problems often requires the incorporation of additional genetic markers. For instance, the ITS2 (Ribosomal Internal Transcribed Spacer 2) and EF1-α (Elongation Factor 1-α) genes, and even microsatellites, have proven to be relatively effective in discriminating among closely related species and/or characterize populations of Tephritidae, including members of the *fraterculus* complex, and other pestiferous insects [26, 27, 28, 29, 30]. Linear and geometric morphometric analyses are also useful for discriminating 3rd instar larvae and regional morphotypes across the *fraterculus* complex [24, 31, 32, 33], and at ruling out conspecificity of putative members at smaller geographic scales [34, 35, 36, 37]. Therefore, implementing integrative methods is necessary to delimit species when DNA barcoding data reveals the presence of members of the *fraterculus* group in a particular context [38].

## Conclusions

**Fig 3:**
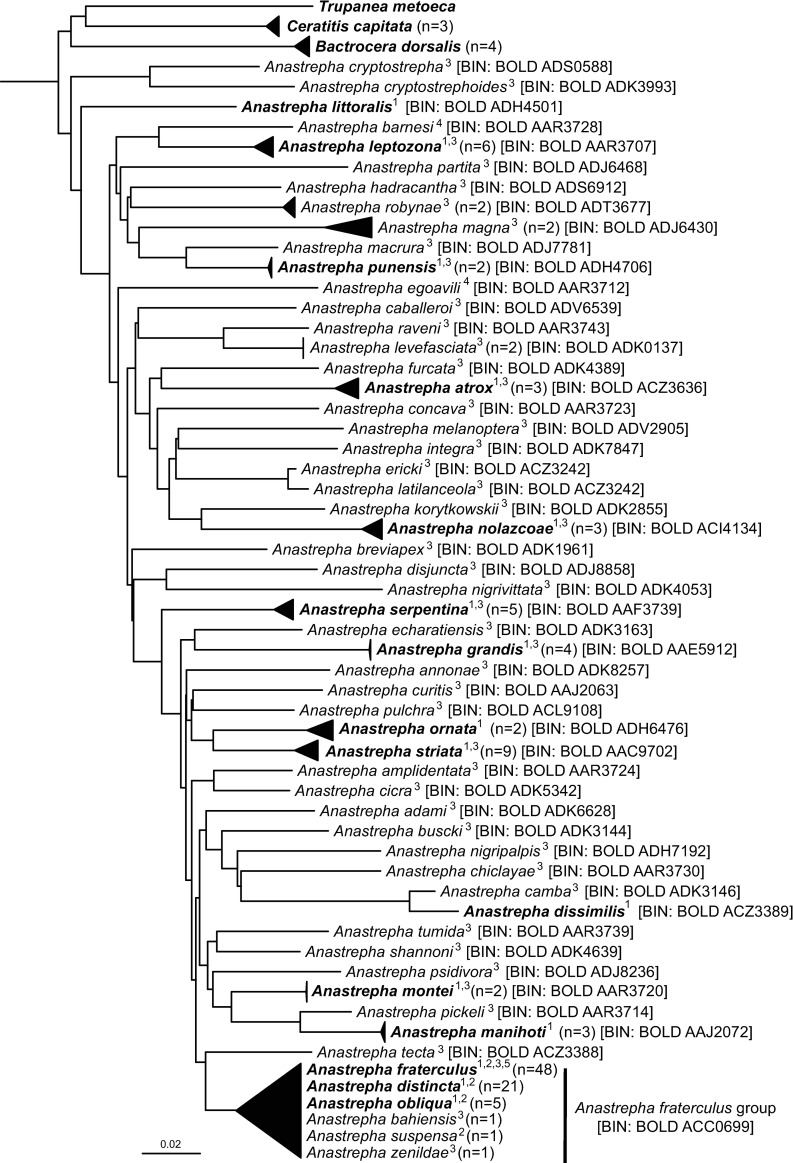
DNA barcoding tree (K2P) of 56 Peruvian species of *Anastrepha*. Superscripts indicate the origin of the sequence(s) representing each taxa: ^1^this study (highlighted in bold); ^2^Scally et al. (2016); ^3^Mengual et al. (2017); ^4^Barr et al. (2018); ^5^Armstrong & Ball (2005). Branches with ≥ 2 specimens were collapsed for simplicity (n = number of specimens in collapsed branch). Square brackets besides each terminal taxon indicates its BIN number according to the Barcode of Life Data System (BOLD). All BINs represent a single species, except for *A*. *ericki* and *A*. *latilanceola* (*mucronata* species group) and all tested members of the *A*. *fraterculus* group. Maximum and mean values of genetic divergence for this combined dataset were 18.5 (*A*. *pickeli* vs. *A*. *cryptostrephoides*) and 8.47%, respectively. This analysis was conducted on MEGA X on a final dataset of 159 ingroup and 8 outgroup sequences (658 nucleotide positions), and represents the most comprehensive DNA barcoding tree profile of *Anastrepha* spp. from Peru to date.

Our preliminary assessment of the efficacy of the DNA barcoding tool at discriminating most Peruvian samples of *Anastrepha* species, except notably for those in the *fraterculus* group (Fig 3). Although morphology-based identification is possible for adult females of species sharing the same barcode, it is not feasible for larvae or adult males of many species). The matter is complicated for members of the *A*. *fraterculus* complex, which comprises a yet uncertain number of cryptic species whose identification through COI-based DNA barcoding is currently not possible [19]. However, given the paramount economic importance of these flies across the world [39], introducing complementary methodologies is needed to discriminate among these species, especially with regard to the cryptic members of the *fraterculus* complex and its morphotypes occurring within Peru [32]. Similarly, expanding geographic and taxonomic coverage (and thus genetic diversity), as well as sampling across host crops, could help to improve the effectiveness of molecular identification through DNA barcodes as well as the ability to distinguish among regional lineages with distinct ecological preferences that may differ in their invasive potential. DNA barcoding could also assist the implementation of pest control actions or identifying pestiferous species during routine quarantine inspection at ports of entry (e.g. [25, 40, 41, 42].

The need for accurate taxonomic identification will only increase as invasive agricultural pests are predicted to increase mobility due to climate change [43, 44]. Therefore, incorporating molecular-based identification methods into biosecurity protocols is becoming a global priority [16, 45]. Despite the problematic nature of species complexes, which limit the application of DNA barcoding technology for taxonomic identification [46], the implementation of this tool in the Peruvian context has nevertheless potential to become a useful diagnostic tool for crop protection and pest control.

## Supporting information

S1 Table Spreadsheet containing voucher data. (XLSX)

S1 File Alignment of 167 COI sequences of 56 species of *Anastrepha* from Peru and outgroups (Fasta format). Taxon label annotation: BOLD record code**|**species name**|**COIN5P**|**Genbank Accession Number.
(FAS)
